# Flowering Stage and Daytime Affect Scent Emission of *Malus* *ioensis* “Prairie Rose”

**DOI:** 10.3390/molecules24132356

**Published:** 2019-06-26

**Authors:** Junjun Fan, Wangxiang Zhang, Donglin Zhang, Guibin Wang, Fuliang Cao

**Affiliations:** 1 College of Forestry, Nanjing Forestry University, Nanjing 210037, China; 2 Co-Innovation Center for Sustainable Forestry in Southern China, Nanjing Forestry University, Nanjing 210037, China; 3 Department of Horticulture, University of Georgia, Athens, GA 30602, USA

**Keywords:** crabapple, flower fragrance, emission rule, SPME-GC/MS

## Abstract

Flowering crabapple is an important ornamental flower. It is vital to understand the floral scent properties and the associated release dynamics for carrying out fragrant flower breeding or floral regulation of crabapple. Static headspace solid-phase microextraction coupled with gas chromatography-mass spectrometry was used to detect the volatile compounds in *Malus* *ioensis* “Prairie Rose” flowers at different flowering stages and at different day-night time. The results showed that methylheptenone, phenylethanol, geranylacetone, 2-(4-methoxyphenyl)ethanol, α-cedrene were the major compounds in *M. ioensis* “Prairie Rose”, but the compounds released during different stages and different day-night time were significantly different (*P* < 0.0001). A total of 25 volatile compounds were identified from the four flowering stages. The floral scents in the initial and flowering stages were the most similar (dissimilarity 0.21). The main compounds in these two stages were geranylacetone and methylheptenone, and the contents of geranylacetone and phenylethanol were positively correlated with the flowering stages. From the bud stage to the end of flowering, the total amount of volatile compounds released showed an initial increase followed by a decrease and the amounts of compounds released during the initial flowering stage were the highest. The aliphatic and benzenoids content was significant higher in the daytime than at night. A total of 15 compounds were detected in the five time periods. Methylheptenone and phenylethanol were particularly released in the 10:00–12:00 and 15:00–17:00 time periods. There were only three common compounds among the five time periods and the types of flower volatiles released during the daytime were obviously higher than those released at night. From the nocturnal to diurnal, the amount of flower volatiles released first increased, then decreased, and the release reached a peak between 10 am and 12 noon, which was consistent with the pollination biological characteristics of *Malus* flowers. Our findings are important for understanding the mechanism of insect visits to crabapple and the regulation of crabapple flower scent.

## 1. Introduction

Floral scent compounds are secondary metabolites released by plant flowers. These are mainly volatile compounds with low molecular weight (generally less than 300 Dalton), such as terpenoids, benzenes/phenylpropanoids, aliphthics, and some nitrogen- and sulfur-containing compounds [1]. The floral scent is an important expression signal for plants to induce insect pollination, as well as an important quality indicator for evaluating ornamental plants and cut flowers [2]. Studies had shown that floral scent had a greater influence on consumers than flower color and shape [3]. However, most of the common flower-breeding goals are concentrated on flower shape, flower color, and flowering time, with the result that flowers are not bred for fragrance [4], and many cultivated flowers have gradually lost their aroma [5].

Flowering crabapple (*Malus*) is an important ornamental flower in the northern temperate zone [6]. China is the distribution center of *Malus* [7,8], but the long-term and widespread use of cultivars is mainly limited to a few species, such as *M. halliana*, *M. micromalus* and *M. hupehensis.* Many precious crabapple germplasm resources have not been fully applied. The traditional view in China is that flowering crabapple flowers is beautiful but not fragrant [9]. However, long-term natural selection and artificial breeding have resulted in more than 700 crabapple cultivars worldwide [10,11,12], most of which are aromatic [9]. Zhao et al. [9] measured the floral compounds of 17 crabapple cultivars and found that the main compounds were 3-methyl-1-butanol, octene, benzyl alcohol, 3-methyl-4-oxo-penta acid, and heptane. Li [13] examined the floral and leaf compounds of *M*. *baccata* and found that the compounds responsible for the floral scent were mainly butylated hydroxytoluene, heptadecane, hexadecane, α-farnesene and β-octene. Fan et al. [14] used electronic nose and gas chromatography-mass spectrometry to measure the different aroma intensities of crabapple and initially explored the relationship between the scent compounds and the electronic nose. At present, research on crabapple floral scent is far behind research on the flowering period [15], flower color [16,17], flower shape [10] and pollen [18], and there are no reports on the release characteristic of crabapple floral scent. In fact, a plant often has more than one dozen to hundreds of volatile compounds, and these compounds and the associated level of scent emission change with flower age, endogenous circadian rhythms, pollination status, and environmental conditions (such as moisture, light, and temperature) [19,20,21]. There are no two floral scents that are exactly the same, even among plants with similar flower shape and color, due to the large diversity of volatile compounds and their relative abundance and interaction [22,23]. Thus, when carrying out fragrant flower breeding or floral regulation, even essential oil extraction, it is vital to understand the floral scent properties of a plant and the associated release dynamics.

We used *M*. *ioensis* “Prairie Rose” as materials, which has strong scent and beautiful rose-like flower shape, to compare the difference of volatile compounds of flower at different flowering stages and day-night times using headspace solid-phase microextraction and gas chromatography-mass spectrometry (HS-SPME-GC-MS). Our objective is to explore the floral scent characteristics and release dynamics of *M*. *ioensis* “Prairie Rose”.

## 2. Results

### 2.1. Volatile Compounds Emission Characteristic of Different Flowering Stages

#### 2.1.1. Identification and Comparison of Volatile Compounds of Flowers at Different Flowering Stages

**Figure 1:**
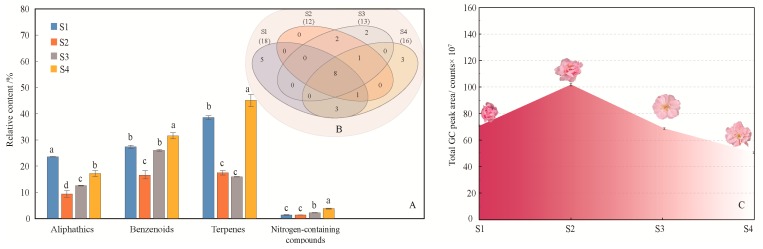
Generation of volatile compounds from *M*. *ioensis* “Prairie Rose” flowers during flowering. (**A**) Compounds of different chemical classes among the four flowering stages. S1 bud stage; S2 initial flowering stage; S3 flowering stage; and S4 end of flowering stage. The same letters above different color column mean there is no significant difference in the same chemical class among four flowering stages (*P* > 0.05), and different letters indicate significant difference (*P* < 0.05). (**B**) Venn diagram indicating the similarities and differences in total volatile compounds among the different flowering stages. The numbers in related overlapping areas indicate the compounds shared between the different flowering stages. (**C**) Volatile compound emissions from *M*. *ioensis* “Prairie Rose” flowers during flowering.

A total of 25 volatile compounds were identified from the four flowering stages of *M. ioensis* “Prairie Rose” (Table 1, supplementary Figure S1), and the compounds released during these were significantly different (*P* < 0.0001). Classification revealed that the main chemical categories of flower scent in the four flowering stages were terpenes, benzenoids and aliphatics (Figure 1A), but the main volatile compounds were not identical in these flowering stages (Table 1). Concretely, the main compounds in the bud stage (S1) were 2-(4-methoxyphenyl)ethanol (16.36%), methylheptenone (16.56%) and α-cedrene (11.71%). The main compounds in the initial flowering stage (S2) were geranylacetone (10.70%) and methylheptenone (6.92%). The main compounds of the flowering stage (S3) was phenylethanol (10.60%), geranylacetone (9.64%) and methylheptenone (8.08%). The main compounds at the end of flowering (S4) were 2-(4-methoxyphenyl)ethanol (16.36%), methylheptenone (11.72%), α-cedrene (12.41%), geranylacetone (10.19%), and phenylethanol (7.51%). Interestingly, phenylethanol released the most at flowering stage. The main compounds in the bud stage and at the end of flowering were similar and their proportions were comparable (Table 1). There were eight common compounds in the four flowering stages (Figure 1B, Table 1). Although the numbers of compounds (18) and specific compounds (5) released during the bud stage were the highest, the amounts of compounds released during the initial flowering stage were the highest. From the bud stage to the end of flowering, the total amount of volatile compounds released showed an initial increase followed by a decrease (Figure 1C).

#### 2.1.2. Difference Analysis of Flower Volatiles Based on Bray-Curtis Dissimilarity Analysis and Principal Component Analysis (PCA)

Bray-Curtis dissimilarity analysis is often used to compare the degree of dissimilarity between two samples [26]. The larger the value, the greater the difference between two subjects (low similarity). For all identified compounds, the dissimilarity among the four flower stages ranged from 0.21 to 0.52 (Table 2). The floral scents in the initial and flowering stages were the most similar (dissimilarity 0.21), and the floral scents in the bud stage and at the end of flowering were also comparable (dissimilarity 0.27).

**Figure 2:**
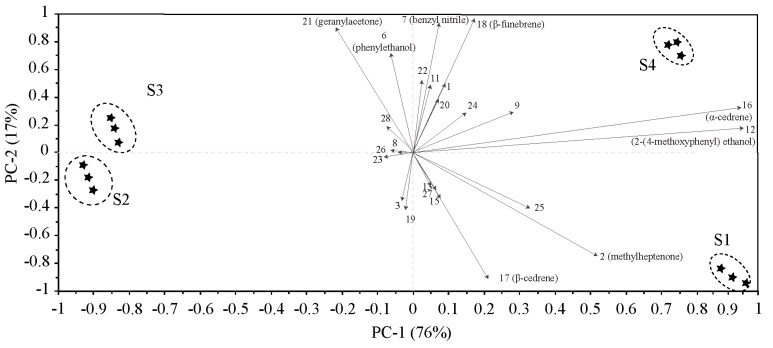
Principal component analysis (PCA) biplot based on the volatile compounds released from *M*. *ioensis* “Prairie Rose” flowers during flowering, showing correlations with volatile compounds (numbers correspond to those in Table 1). S1 bud stage; S2 initial flowering stage; S3 flowering stage; and S4 end of flowering stage.

To further analyze the contribution of different compounds to the floral variation among the four flowering stages, principal component analysis (PCA) was performed based on the 25 compounds (Table 1, Figure 2). The results show that the total contribution of the first principal component (PC1) and the second principal component (PC2) reached 93.0%. Compounds with high scores on PC 1 comprised α-cedrene and 2-(4-methoxyphenyl)ethanol, which were obviously positively related to the bud stage and the end of flowering stage. Compounds that had high positive scores on PC 2 included phenylethanol and geranylacetone, which were highly positively correlated with the flowering stage. The four flowering stages were distributed in four coordinate quadrants without overlapping, which indicated that the compounds of floral scent differed throughout the whole flower development.

### 2.2. Diurnal and Nocturnal Release Rhythm of M. ioensis “Prairie Rose”

#### 2.2.1. Identification and Comparison of Volatile Compounds at Different Times

Table 1 and supplementary Figure S2 show the composition and release rate of *M. ioensis* “Prairie Rose” flowers during the day and night. A total of 15 compounds were detected in the five time periods, and the composition of each time was significantly different (*P* < 0.0001). 

**Figure 3:**
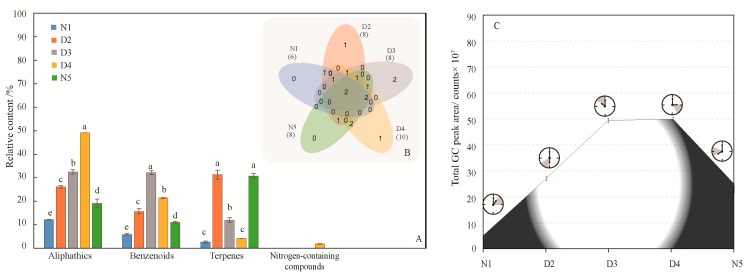
Generation of volatile compounds from *M*. *ioensis* “Prairie Rose” flowers during the day and night. (**A**) Compounds of different chemical classes during day and night. N1 1:00–3:00, D2 6:00–8:00, D3 10:00–12:00, D4 15:00–17:00 and N5 20:00–22:00. The same letters above different color column mean there is no significant difference in the same chemical class among different time periods (*P* > 0.05), and different letters indicate significant difference (*P* < 0.05). (**B**) Venn diagram indicating the similarities and differences in total volatile compounds among the different flowering stages. The numbers in related overlapping areas indicate the compounds shared between the different times. (**C**) Volatile compounds emission from *M*. *ioensis* “Prairie Rose” flowers during day and night.

According to the classification of compounds (Figure 3A), except for 1:00–3:00 and 15:00–17:00 (benzenoids and aliphatics), the chemical categories at the three other times were mainly benzenoids, terpenes, and aliphatics, but the main compounds were not identical in five time periods. The main compounds released from 1:00–3:00 was methyl hexadecanoate; the main compounds released from 6:00–8:00 were α-pinene (19.89%), methylheptenone (14.65%) 2-(4-methoxyphenyl)ethanol (9.16%) and α-cedrene (9.10%); the main compounds released from 10:00–12:00 were 2-(4-methoxyphenyl)ethanol (21.99%), methylheptenone (11.49%), dodecane (9.65%) and phenylethanol (8.67%); the main compounds released from 15:00–17:00 were methylheptenone (31.55%) and 2-(4-methoxyphenyl)ethanol (7.84%); and the main compounds released from 20:00–22:00 were α-pinene (28.54%) and 2- (4-methoxyphenyl) ethanol (8.09%). The aliphatic and benzenoids content were significant higher in the daytime than at night. In particular, phenylethanol was released only during the day time (Table 1). There were only two common compounds among the five time periods (Figure 3B), and the types of flower volatiles released during the daytime were significantly higher than those released at night (*P* < 0.0001). Over the nocturnal, diurnal, nocturnal period, the amount of flower volatiles released first increased and then decreased, and the release reached a peak between 10 am and 15 pm (Figure 3C), which is consistent with the pollination biological characteristics of *Malus* flowers. 

#### 2.2.2. Difference Analysis of Volatiles in Different Flower Parts Based on Bray-Curtis Dissimilarity Analysis and Principal Component Analysis 

Bray-Curtis dissimilarity analysis showed that the difference in the volatile compounds at different times ranged from 0.44 to 0.65 (Table 3), which indicates that the floral release compounds of *M*. *ioensis* “Prairie Rose” changed constantly during the day and night, but approximately half of the compounds in an adjacent time period were similar.

**Figure 4:**
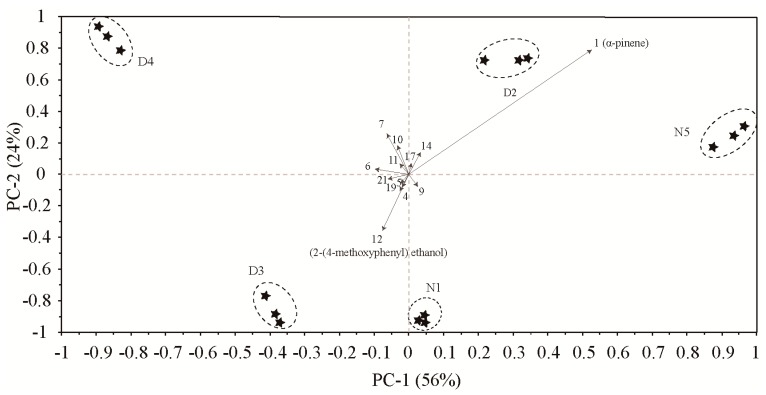
PCA biplot based on the volatile compounds of *M*. *ioensis* “Prairie Rose” flowers during the day and night, showing correlations with volatile compounds (numbers correspond to those in Table 1). N1 1:00–3:00, D2 6:00–8:00, D3 10:00–12:00, D4 15:00–17:00 and N5 20:00–22:00.

To further analyze the contribution of different compounds to the differences in floral scent release over time, we performed PCA (Figure 4) based on 15 compounds (Table 1). The results showed that the total contribution of PC1 and PC2 reached 80%. α-pinene had high positive scores on PC 1, which were highly positively correlated with the 1:00–3:00 and 20:00–22:00 time periods. Compounds with high negative scores on PC 2 contained 2-(4-methoxyphenyl)ethanol, which was highly positively related to the 10:00–12:00 time period. The PCA biplot did not overlap among the five periods indicated that the compounds of floral scent differed throughout the day and night. 

### 2.3. Floral Scent Properties of M. ioensis “Prairie Rose”

The compounds with a lower odor detection threshold (ODT < 0.01 ppm) and higher relative content in the *M. ioensis* “Prairie Rose” flowers were phenylethanol, geranylacetone, and methylheptenone, which were also strongly released during the day (Table 1). Phenylethanol and geranylacetone have a rose-like aroma; methylheptenone has a woody odor. It suggested that the floral characteristics of *M. ioensis* “Prairie Rose” maybe a combination of rose and woody (Table 1).

## 3. Discussion

### 3.1. Flower Characteristics of M. ioensis “Prairie Rose”

Plant aroma is the objective form of the quality and quantity of aromatic compounds in space. Fragrant compounds of different plants flower are different, different proportions of aromatic compounds interact with each other, and some specific compounds even form a unique flower scent of each plant [27,28]. The main volatile compound of the famous fragrant flower, *Osmanthus fragrans*, were linalool, α-ionone, c-decalactone and hexadecanoic acid using SPME-GC/MS method [29]. The major compounds of *Lagerstroemia caudata* flowers were *n*-hexadecanoic acid *trans*-geranylacetone, heptadecane, and dibutyl phthalate [30]. Even different varieties of lilac had different floral components [31]. At present, more than 2000 flower fragrance compounds of nearly 100 plant families have been identified. Many studies have shown that benzenoids are the main compounds of Rosaceae flowers [1,32,33]. Our study showed that the main compounds of *M. ioensis* “Prairie Rose” are also benzenoids, which is consistent with the findings presented by Fan [14], Zhao et al. [9] and Li [13]. However, Zhao et al. [9] and Li [13] found that one of main benzenoids compound was benzyl alcohol, whereas it was phenylethanol in our study. Benzyl alcohol and phenylethanol were the two different metabolites of phenylalanine [34]. Benzyl alcohol have a fruit-like aroma, but phenylethanol have a rose-like aroma and is one of the main compounds of roses [35]. In fact, the main compounds of *M. ioensis* “Prairie Rose”, *M*. “Brandywine” and *M*. “Klehm’s Improved Bechtel”, which floral morphology are all similar to rose flower, were similar but different from other crabapple germplasms (data unpublished). Moreover, phenylethanol, geranylacetone (rose-like aroma), and methylheptenone (woody aroma) were also the main characteristic aroma compounds of these three cultivars, which had lower aromatic threshold and higher relative content. The lower the threshold, the stronger the aromatic intensity and the greater the contribution to the floral aroma characteristics [36]. Therefore, it can be assumed that the floral characteristics of *M. ioensis* “Prairie Rose” and other two cultivars are a combination of rose and woody. These volatile compounds were mainly released during the daytime and was not detected or was rarely detected at night. In addition, when *M. ioensis* “Prairie Rose” flower is in flowering and in diurnal time, the relative contents of phenylethanol and geranylacetone were higher than that in other flower stage or diurnal time. This indicates that when *M. ioensis* “Prairie Rose” is in flowering, the rose scent will be more intense.

### 3.2. The Release Dynamics of the Floral Scent of M. ioensis “Prairie Rose”

The release characteristics of aromatic compounds in different plants during flower development may be different. Studies have found that in order to maximize pollination opportunities, most flowers often begin to produce aroma when they are ready to receive pollen and the insects involved in pollination are most active [37,38]. When the flowers are completely open, the highest number of different types and maximum amounts of volatiles are released, and after pollination, the aromas gradually weaken until they disappear [39]. In the current study, in the flowering stage of *M. ioensis* “Prairie Rose”, the flower compounds in the bud stage and the late flowering stage were more similar than that in other stages (dissimilarity 0.27), and the flower compounds in the initial flowering and flowering periods were the most similar (dissimilarity 0.21). α-Cedrene, 2-(4-methoxyphenyl)ethanol, geranylacetone, and phenylethanol, played a major role in the four flowering stages.

The type of plant pollinator also affects the composition of the diurnal and nocturnal floral scent release. In general, a flower that is mainly pollinated by day pollinators (such as bees) will produce more scent during the day; by contrast, flowers that rely mainly on moths for pollination will produce more volatile compounds at night [40,41,42]. *Malus* species are mainly pollinated by bees, and flowers are generally in the best pollination state at approximately 10 am [43]. Our results showed that the floral scent release reached its peak between 10:00–12:00, which is consistent with the pollination biological characteristics of this species. The main volatile compounds of *M. ioensis* “Prairie Rose” flowers, i.e., methylheptenone, phenylethanol, and 2-(4-methoxyphenyl)ethanol, were strongly released during the day to attract daytime pollinating insects.

### 3.3. Limitations of Floral Collection Methods 

An approach to detect flower aroma rapidly and accurately has always been a problem for researchers and is also an important reason floral research lags far behind flower color and flower shape research [19,44,45]. The current mainstream method for determining flower volatiles is HS-SPME-GC/MS [46]. In addition, infrared spectroscopy, nuclear magnetic resonance techniques, distillation and extraction methods are often used to detect plant volatiles [47]. However, all current methods are difficult to collect 100% floral compounds because it is difficult to ensure that one adsorption medium adsorbs or extracts all volatile molecules, and there is no guarantee that the adsorption rate or extraction rate of each medium is equal. Floral compounds of known structure and molecular formula are easier to detect than other unknown substances. It is expected that with the improvement of the methods used to detect floral substances, an increasing of floral substances will be discovered and identified. The solid-phase microextraction static headspace sampling used in this study has the advantage of the enrichment of volatiles in a closed sampling space, suitable for those with low volatility. However, when sampling times are too long, humidity and a lack of gas exchange can affect normal physiological processes and affect the volatiles released by the flower [19]. Therefore, the measurement of the floral circadian rhythm that we perform outdoors may be affected by external temperature, sunlight, humidity, etc., and the type and content of the detected compounds are lower than those in an indoor, stable environment. In addition, adsorption rate of SPME fiber for each compound is unequal, it can be quantified by chemical compound standards. This is beneficial to the accurate quantitative analysis of aroma compounds rather than using the relative content, which can be further studied.

## 4. Materials and Methods

### 4.1. Plant Materials

*M. ioensis* “Prairie Rose” flowers were obtained from the National Crabapple Germplasm Resource database (Jiangdu District, Yangzhou City) (longitude 119°55′ E, latitude 32°42′ N). The site experiences a northern subtropical monsoon climate and four distinct seasons. The annual average temperature is approximately 14.9 °C, average rainfall is approximately 1000 mm, and the frost-free period is approximately 320 days. There were a total of 30 *M. ioensis* “Prairie Rose” plants, 8 years old, which were planted at a spacing of 2 × 3 m.

### 4.2. Experimental Design

Two experiments were established using a single factor design to explore the emission characteristics of *M*. *ioensis* “Prairie Rose” based on different flowering stages and times of the day and night.

**Figure 5:**
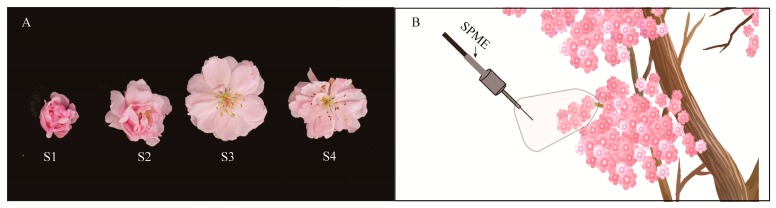
Schematic diagram of sample collection of *M*. *ioensis* “Prairie Rose” flowers. (**A**) Typical samples of four flowering stages. S1: large bud stage (the buds were swollen, the male and female stamens were about to appear); S2: early flowering stage (the flowers were about to open, and the male and female stamens were exposed); S3: flowering stage (flowers were completely open and stigma and anthers had fresh, bright colors); S4: the end of flowering stage (stigma and anthers were dry, petals were not dry). (**B**) Real-time sampling on living plants from 1:00 to 22:00.

Experiment 1: The release of volatile compounds from *M*. *ioensis* “Prairie Rose” flowers in different flowering stages. Sample collection: Three individual plants with similar growth were selected, and five inflorescences per plant in the large bud stage (S1) (the buds were swollen, the male and female stamens were about to appear), early flowering stage (S2) (the flowers were about to open, and the stamens and pistil were exposed), flowering stage (S3) (flowers were completely open and stigma and anthers had fresh, bright colors), and at the end of flowering (S4) (stigma and anthers were dry, petals were not dry) were collected (Figure 5A). The inflorescences from the different stages were separately placed in deionized water before being transported to Nanjing Forestry University (Nanjing, China) where they were maintained at room temperature (25 ± 1 °C). Sample preparation: Approximately 4 g of de-stalked flowers at each stage was weighed during 10:00–12:00, placed in a 200-mL headspace bottle, sealed, and placed in a room at 25 °C for 30 min for collection and identification of floral scent. The sampling was repeated three times. 

Experiment 2: The diurnal and nocturnal release of *M*. *ioensis* “Prairie Rose” floral scent. In situ sampling of live plants occurred at 1:00–2:00, 6:00–7:00, 10:00–11:00, 15:00–16:00 and 20:00–21:00. The same bouquet of blooming flowers (5–7 flowers, approximately 4 g) was sampled, and the flowers were placed in a sampling bag (Reynolds, 406 mm × 444 mm, Richmond, VA, USA) on (Figure 5B). The sampling bag was sealed, equilibrated for 30 min to accumulate volatiles, and then subjected to a portable SPME equipped with a 65 μm PDMS-DVB (polydimethylsiloxane/divinylbenzene) fiber (Supelco, Bellefonte, PA, USA; The instrument is capable of storing adsorbed volatile chemical compounds for up to 2 weeks at low temperatures (<0 °C) with no significant component loss [48].) for 30 min. Consequently, the entire collection period was 1:00–3:00 (N1), 6:00–8:00 (D2), 10:00–12:00 (D3), 15:00–17:00 (D4) and 20:00–22:00 (N5). A blank sampling bag was used as a control. The sampling was repeated three times. After sampling, the portable SPME was stored in a dry cooler (−5 °C) and then returned to the laboratory for component identification.

### 4.3. SPME-GC-MS Analysis

The portable SPME equipped with a 65 um PDMS-DVB fiber (Supelco, Bellefonte, PA, USA) was inserted into the headspace of the capped vial to absorb volatile compounds of flower for 0.5 h at 25 °C. An empty capped vial was used as a blank control. The scent of all samples in experiment 2 and 3 was extracted at the same time, and the samples were injected into the GC in a random order.

A GC system (Thermo Fisher Scientific, Waltham, MA, USA) equipped with a DB-5MS fused silica capillary column (30 m × 0.25 mm i.d.; 0.25 μm film thickness; 5% phenylmethyl siloxane; Agilent Technologies, Santa Clara, CA, USA) was used for compounds identification. Following volatile compounds extraction by SPME, the fiber of the SPME was inserted into the GC injector port in splitless mode for desorption at 250 °C for 5 min. Helium was used as the carrier gas at a flow rate of 1.0 mL·min^−1^. The temperatures of the transfer line and ion source were 230 and 210 °C, respectively. The temperature program of column oven was as follows: 50 °C for 1 min, increasing at 4 °C·min^−1^ to 120 °C and then held for 1 min, followed by an increase at 1.5 °C·min^−1^ to 140 °C, and then increase at 12 °C·min^−1^ to 230 °C, with no hold. The electron ionization potential of the mass detector was 70 eV, and the scan range was from 35 to 450 amu. Linear retention indices (LRI) of the volatile compounds were calculated using an alkane series standard (C7–C30) (Sigma, St. Louis, MO, USA) under the same conditions. In our study, no standard was used, and the identifications are tentative, based only on MS similarity and LRI. Each sample had three replicates, and mean values with relative standard deviations (mean standard deviation, %) were reported.

### 4.4. Data Analysis 

Identification of volatile compounds: Compounds identification was performed by comparing the mass spectra with the National Institute of Standards and Technology (NIST) 12th library (probability > 75%). Meanwhile, these compounds were verified by some published databases, including plant scent database (SuperScent: http://bioinf-applied. charite.de/superscent/), chemistry database (PubChem: http://pubchem.ncbi.nlm.nih.gov/); LRI database (NIST Chemistry WebBook: https://webbook.nist.gov/chemistry/cas-ser/). 

Calculation of volatile compounds content: The relative content of each compound was calculated by normalizing the peak area (Xcalibur 3.1 (Thermo Fisher Scientific, Waltham, MA, USA)).

PCA was performed using Unscrambler v. 10.4 (CAMO, Oslo, Norway). Variance analysis of floral compounds at different flowering stages and during different times was performed by SPSS v. 19.0 (IBM Corp., Armonk, NY, USA). Bray-Curtis dissimilarity analysis was performed using R 3.4.3.

## 5. Conclusions

In this study, static headspace solid-phase microextraction coupled with gas chromatography-mass spectrometry was used to detect the volatile compounds in *M*. *ioensis* “Prairie Rose” flowers at different flowering stages and at different times of the day and night. Methylheptenone, phenylethanol, geranylacetone, 2-(4-methoxyphenyl)ethanol, α-cedrene were the major compounds in *M*. *ioensis* “Prairie Rose”. Phenylethanol, geranylacetone, and methylheptenone were the floral dominant compounds of “Prairie Rose”, which smelled like rose and wood. Different flowering stages and day-night time had great influence on the release of flower scent components of “Prairie Rose”. During the day, the number of characteristic aromas released was maximized, especially between 10:00–12:00, when the flowers from the initial flowering stage to the flowering stage were in the optimal pollination period. Our findings are important for understanding the mechanism of insect visits to crabapple and the regulation of crabapple flower scent.

## Figures and Tables

**Table 1:** Volatile compounds detected in *M*. *ioensis* “Prairie Rose” flowers at different flowering stages and during the day and night using SPME-GC-MS. Please note that all identifications are tentative since no direct comparison with authentic standard compounds was made.

No.	Time/min	Compounds	CAS	LRI	LRI ^a^	Probability/%	ODT ^b^/ppm	Odor Characteristic ^c^	Relative Content/%								
									Bud	Initial Flowering	Flowering	End Flowering	1:00–3:00	6:00–8:00	10:00–12:00	15:00–17:00	20:00–22:00
1	6.93	α-pinene	80-56-8	877	907	87.5–92.1	0.12–1.01	fruity, sweet, pine	--	--	--	2.62 ± 0.21	--	19.89 ± 1.12b	--	--	28.53 ± 1.38a
2	9.43	methylheptenone	110-93-0	937	942	81.6–93.4	0.0189	mushroom, earthy, woody	16.36 ± 0.23a	6.92 ± 0.9d	8.08 ± 0.26c	11.72 ± 0.61b	2.35 ± 0.24d	14.65 ± 0.97b	11.5 ± 1.06c	31.54 ± 1.34a	--
3	9.92	4-methylanisole	104-93-8	972	989	84.8–91.9	0.0029		1.83 ± 0.06a	1.39 ± 0.29c	1.48 ± 0.09b	--					
4	12.15	linalool	78-70-6	1079	1082	83.8	0.0015	floral, woody, fresh					--	--	3.61 ± 0.34	--	--
5	12.32	nonanal	124-19-6	1098	1098	76.4–89.2	0.0031	floral, sweet, citrus	1.46 ± 0.13	--	--	--	4.83 ± 0.19b	--	5.6 ± 0.5b	6.88 ± 0.76a	7.34 ± 0.66a
6	12.58	phenylethanol	60-12-8	1165	1150	78.2–91.5	0.012	rose, honey, fragrant	6.02 ± 0.33c	3.76 ± 0.2d	10.59 ± 0.68a	7.51 ± 0.48b	--	5.36 ± 0.9b	8.68 ± 1.5a	5.21 ± 0.13b	--
7	12.77	benzyl nitrile	140-29-4	1178	1160	82.8–93.6	1	herbal, floral	2.83 ± 0.06c	2.81 ± 0.31c	5.18 ± 0.24b	7.41 ± 0.47a	--	--	--	7.24 ± 0.7a	2.62 ± 0.51b
8	15.57	cuminaldehyde	122-03-2	1212	1214	94.7	0.14	sharp, woody	--	--	1.2 ± 0.09	--					
9	15.6	dodecane	112-40-3	1231	1200	83.2–86.5	0.77	fusel-like	3.06 ± 0.09b	--	--	4.4 ± 0.37a	5.02 ± 0.32c	7.59 ± 0.53b	9.64 ± 1.64a	4.25 ± 0.71c	7.49 ± 0.39b
10	15.75	decanal	112-31-2	1219	1220	79.9–95.0	0.003	green, floral, lemon,					--	--	--	5.03 ± 0.43a	2.48 ± 0.39b
11	18.87	(2-nitroethyl) benzene	6125-24-2	1321	1304	80.6–91.2	0.002	sweet, floral, spicy	1.36 ± 0.19c	1.41 ± 0.03c	2.23 ± 0.24b	3.83 ± 0.18a	--	--	--	1.84 ± 0.15	--
12	21.49	2-(4-methoxyphenyl) ethanol	702-23-8	1381	1374	79.8–87.9	--	fresh citrus	16.56 ± 0.56a	3.58 ± 0.43c	5.25 ± 0.62b	16.36 ± 1.21a	6.56 ± 0.32d	9.16 ± 0.22b	21.96 ± 1.08a	7.84 ± 0.41c	8.1 ± 0.41c
13	21.79	texanol	77-68-9	1380	1380	90.3	--		1.26 ± 0.04	--	--	--					
14	22.19	(*Z*)-3-hexenyl hexanoate	31501-11-8	1389	1380	84.9–89.5	0.0052	fruity, green					2.66 ± 0.45b	9.1 ± 0.25a	--	--	--
15	22.32	β-elemene	515-13-9	1443	1429	89.4	--	herbal	1.70 ± 0.20	--	--	--					
16	23.48	α-cedrene	469-61-4	1453	1444	88.4–90.2	0.00003	woody	11.71 ± 0.36a	--	--	12.41 ± 1.29a					
17	23.82	β-cedrene	546-28-1	1456	1447	82.6–88.4	0.00003	woody	5.02 ± 0.14	--	--	--	--	2.62 ± 0.59	--	--	--
18	23.96	β-funebrene	79120-98-2	1457	1434	92.6	--		--	--	--	5.00 ± 0.19					
19	24.1	α-ionone	127-41-3	1434	1434	83.3–95.1	0.001–0.006	violet, woody, fruity	4.39 ± 0.5a	3.8 ± 0.56a	3.64 ± 0.09a	2.32 ± 0.16b	--	--	2.61 ± 0.52	--	--
20	24.45	cis-thujopsene	470-40-6	1462	1456	88.2	--		--	--	--	2.05 ± 0.29					
21	25.2	geranylacetone	689-67-8	1456	1456	79.3–89.8	0.06	fresh, floral, rose, fruity	4.92 ± 0.4b	10.7 ± 1.26a	9.64 ± 0.33a	10.19 ± 0.76a			5.77 ± 0.81a	4.16 ± 0.21b	2.24 ± 0.23c
22	27.09	β-ionone	79-77-6	1493	1491	88.1–93.9	0.001–0.006	earthy, woody	1.3 ± 0.09c	1.7 ± 0.03c	2.71 ± 0.08b	3.95 ± 0.74a					
23	27.88	pentadecane	629-62-9	1492	1500	84.6–91.5	0.5	mild green, fusel-like	--	1.16 ± 0.16a	1.03 ± 0.06a	--					
24	28.36	α-farnesene	502-61-4	1496	1496	80.2–87.9	2	woody	1.96 ± 0.37b	1.3 ± 0.14c	--	3.58 ± 0.16a					
25	32.09	cedrol	77-53-2	1528	1573	85.4–90.5	0.00013	sweet	5.42 ± 0.31a	--	--	2.99 ± 0.30b					
26	38.95	octacosane	630-02-4	2802	2800	85.9	--		--	--	1.80 ± 0.12	--					
27	39.14	heneicosane	629-94-7	2082	2100	90.3	--		1.46 ± 0.33	--	--	--					
28	39.58	methyl hexadecanoate	112-39-0	1920	1925	86.8–93.1	>2	faint, sweet	--	1.33 ± 0.16b	1.69 ± 0.02a	1.10 ± 0.12b	20.51 ± 0.95a	5.25 ± 0.23c	--	5.35 ± 0.31c	9.73 ± 0.47b

LRI indicates that linear retention indices of volatile compounds were calculated using an alkane series standard (C7–C30); LRI ^a^ indicates that the data was obtained from National Institute of Standards and Technology (NIST) Chemistry WebBook: https://webbook.nist.gov/chemistry/cas-ser/; ^b^ indicates that the data was obtained from Literature [24]. -- indicates that the data have not been reported by other studies or the compound has not been detected. ^c^ indicates that the odor characteristic of each volatile compound was obtained from Pherobase: http://www.pherobase.com/. Probability was reported by NIST (12th) mass spectra database matching, which reflected the similarity between experimental mass spectra and NIST mass spectra database. Compounds with a matching probability higher than 75% were listed in the Table 1. ODT: The odor detection threshold (ODT) is the minimum concentration of a volatile compound that is perceived by the human olfactory system and is a quantitative representation of the intensity of a fragrance [25]. The relative content with same letters in same color column mean there is no significant difference among different flowering stages, different flower parts or different periods (*P* > 0.05), and different letters indicate significant difference (*P* < 0.05).

**Table 2:** The Bray-Curtis dissimilarity index among different stages of *M*. *ioensis* “Prairie Rose” flowers.

Flowering Stage	S1	S2	S3	S4
S1	0			
S2	0.52	0		
S3	0.52	0.21	0	
S4	0.27	0.48	0.41	0

Note: S1 bud stage; S2 initial flowering stage; S3 flowering stage; and S4 end of flowering stage.

**Table 3:** The Bray-Curtis dissimilarity index of *M*. *ioensis* “Prairie Rose” floral scent during the day and night.

Times of Day	N1	D2	D3	D4	N5
N1	0				
D2	0.65	0			
D3	0.61	0.49	0		
D4	0.63	0.55	0.47	0	
N5	0.60	0.44	0.63	0.59	0

Note: N1 1:00–3:00, D2 6:00–8:00, D3 10:00–12:00, D4 15:00–17:00 and N5 20:00–22:00.

## Supplementary Materials

The following are available online. Figure S1: Total ionic chromatogram of volatile compounds emitted from flowers of *M. ioensis* ‘Prairie Rose’ in different stages. S1 bud stage; S2 initial flowering stage; S3 flowering stage; and S4 end of flowering stage, Figure S2: Total ionic chromatogram of volatile compounds emitted from *M. ioensis* ‘Prairie Rose’ flowers during the day and night. N1 1:00–3:00, D2 6:00–8:00, D3 10:00–12:00, D4 15:00–17:00 and N5 20:00–22:00.
