# Supplementary material for: Flowering Stage and Daytime Affect Scent Emission of Malus ioensis “Prairie Rose”
Source: Molecules. 2019 Jun 26;24(13):2356. doi: 10.3390/molecules24132356 (PMC6650908; doi:10.3390/molecules24132356)
Supplement: Supplementary file 1 [file molecules-24-02356-s001.pdf]

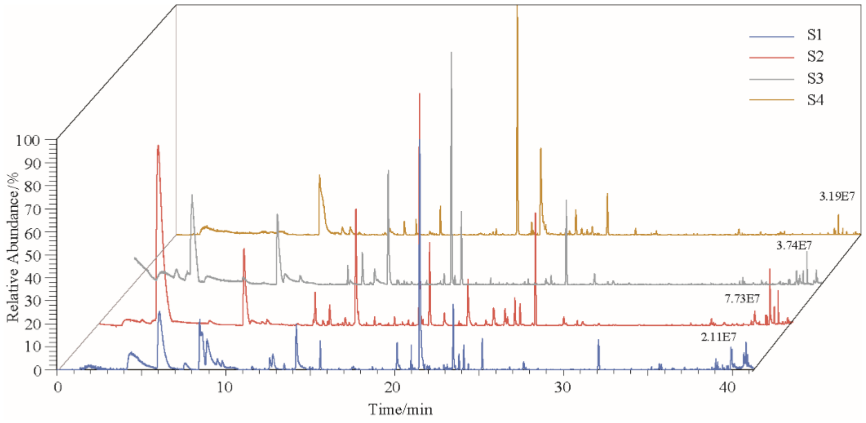

Supplementary Figure S1. Total ionic chromatogram of volatile compounds emitted from flowers of *M. ioensis* 'Prairie Rose' in different stages. S1 bud stage; S2 initial flowering stage; S3 flowering stage; and S4 end of flowering stage.

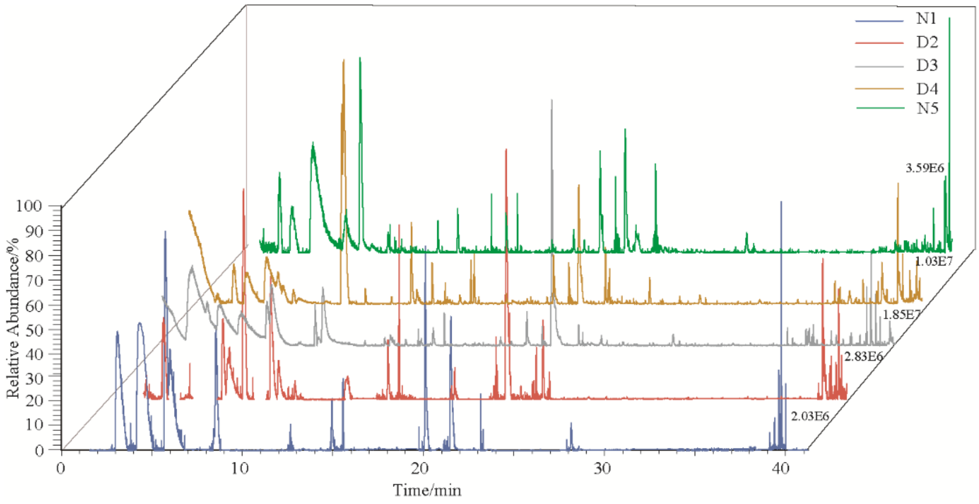

Supplementary Figure S2. Total ionic chromatogram of volatile compounds emitted from *M. ioensis* 'Prairie Rose' flowers during the day and night. N1 1:00–3:00, D2 6:00–8:00, D3 10:00–12:00, D4 15:00–17:00 and N5 20:00–22:00.
